# Are we getting the whole picture? Measuring outcomes using routinely collected data in long term follow-up: an example from BB:2-6

**DOI:** 10.1186/1745-6215-16-S2-O69

**Published:** 2015-11-16

**Authors:** Rebecca Cannings-John, Fiona Lugg, Michael Robling, Gwenllian Moody

**Affiliations:** 1 Cardiff University, Cardiff, UK

## 

The Family Nurse Partnership is an intensive programme of antenatal/postnatal visiting by specially trained nurses to support young pregnant women. The Building Blocks (BB) trial assessed the short-term impact for 1600 teenage mothers and their children from a programme that has existing evidence of longer term benefits. This follow-on study, BB:2-6, will follow-up participants for an additional four years, until their child is six, to determine the longer-term impact of the intervention upon objective indicators of child maltreatment when compared to usually provided health and social care services alone. Follow up will be by linked anonymous data abstraction from the HSCIC and Department for Education, National Pupil Database (NPD).

Whilst this model of linkage offers the possibility for long term evaluation and at a lower cost, what are the associated problems in measuring the proposed study outcomes? This presentation will examine the data sources utilised in the study, and how linkage is carried out by each organisation. An initial assessment of linkage rates and its quality will be reported. As child maltreatment data may also be sourced from LA departments of social services, we will discuss what might be gained and lost for the study in moving from utilising data from this source (i.e. collecting direct from LAs) to the NPD. We will also discuss the consequences for certain analyses such as the Markov Chain Modelling which requires detail about stages of progression through a process and how the quality of the NPD could be validated by sampling local authorities.

